# Molecular genetic and biochemical evidence for adaptive evolution of leaf abaxial epicuticular wax crystals in the genus *Lithocarpus* (Fagaceae)

**DOI:** 10.1186/s12870-018-1420-4

**Published:** 2018-09-17

**Authors:** Chih-Kai Yang, Bing-Hong Huang, Shao-Wei Ho, Meng-Yuan Huang, Jenn-Che Wang, Jian Gao, Pei-Chun Liao

**Affiliations:** 1 School of Life Science, National Taiwan Normal University, Postal address: No. 88, Tingchow Rd. Sect. 4, Taipei, 11677 Taiwan; 2 The Experimental Forest, College of Bio-Resources and Agriculture, National Taiwan University, Taipei, Nantou 55750 Taiwan; 3 Department of Horticulture and Biotechnology, Chinese Culture University, Taipei, 11119 Taiwan; 4 Faculty of Resources and Environment, Baotou Teachers’ College, Inner Mongolia University of Science and Technology, Inner Mongolia, 014010 China

**Keywords:** Adaptive evolution, Leaf epicuticular wax, *Lithocarpus*, Physical defenses, Chemical defenses, Phylogenetic signal

## Abstract

**Background:**

Leaf epicuticular wax is an important functional trait for physiological regulation and pathogen defense. This study tests how selective pressure may have forced the trait of leaf abaxial epicuticular wax crystals (LAEWC) and whether the presence/absence of LAEWC is associated with other ecophysiological traits. Scanning Electron Microscopy was conducted to check for LAEWC in different *Lithocarpus* species. Four wax biosynthesis related genes, including two wax backbone genes *ECERIFERUM 1* (*CER1*) and *CER3*, one regulatory gene *CER7* and one transport gene *CER5*, were cloned and sequenced. Ecophysiological measurements of secondary metabolites, photosynthesis, water usage efficiency, and nutrition indices were also determined. Evolutionary hypotheses of leaf wax character transition associated with the evolution of those ecophysiological traits as well as species evolution were tested by maximum likelihood.

**Results:**

Eight of 14 studied *Lithocarpus* species have obvious LAEWC appearing with various types of trichomes. Measurements of ecophysiological traits show no direct correlations with the presence/absence of LAEWC. However, the content of phenolic acids is significantly associated with the gene evolution of the wax biosynthetic backbone gene *CER1*, which was detected to be positively selected when LAEWC was gained during the late-Miocene-to-Pliocene period.

**Conclusions:**

Changes of landmass and vegetation type accelerated the diversification of tropical and subtropical forest trees and certain herbivores during the late Miocene. As phenolic acids were long thought to be associated with defense against herbivories, co-occurrence of LAEWC and phenolic acids may suggest that LAEWC might be an adaptive defensive mechanism in *Lithocarpus*.

**Electronic supplementary material:**

The online version of this article (10.1186/s12870-018-1420-4) contains supplementary material, which is available to authorized users.

## Background

The evolution of a waterproof epidermis is considered important for plants’ colonization of the land [1]. A waterproof epidermis trait, such as leaf epicuticular wax, is a kind of xeromorphic feature [2] which helps reduce the evapotranspiration rate and allows a plant to adapt to an arid environment [2, 3]. In the case of leaf epicuticular wax, it thickens the boundary layer to reduce evapotranspiration, but its appearance may also reduce the absorption of CO_2_ and reduce photosynthesis efficiency. The partial pressure of CO_2_ is small (0.01~ 0.036%) and the heavier molecular weight of CO_2_ than H_2_O leads to a slower CO_2_ diffusion rate than evapotranspiration rate and so the appearance of leaf epicuticular wax is suggested as an advantageous feature for adapting to arid environments [4]. The accumulation of leaf epicuticular wax is thought to be regulated by light intensity and relative humidity, suggesting that the water regulation of epicuticular wax may be attributed to environmental signals [5]. In addition, the hydrophobic nature of epicuticular wax could decrease the contact area of water, causing water to drain more quickly [6] which, in turn, aids in the removal of dust, bacteria, and epiphytes (i.e. self-cleaning, the Lotus effect). Epicuticular wax may also help maintain the physical integrity of plant leaves by reducing damage caused by pathogens [7]. Therefore, it is suggested as an adaptive indicator of the physical defense of plants [8].

The biosynthesis of epicuticular wax occurs in epidermal cells, in which the very-long-chain-fatty-acids (VLCFAs) are synthesized in endoplasmic reticula. Following this is modification and regulation which synthesizes the precursor of wax. This is then transported to the outer membrane via the ATP-binding cassette (ABC) transporter [9]. Upregulation of wax biosynthetic genes in *Arabidopsis* is a response to the water content of plants in the face of flooding or drought stress [9–12]. Transcriptomic comparisons between the domesticated tomato (*Solanum lycopersicum*) and the desert-adapted accession (*Solanum pennellii*) revealed differential expression in several wax biosynthetic related genes, including the *ECERIFERUM1* (*CER1*), *CER2*, *CER3*, *CER4*, *CER5*-like, *CER6*, *CER7*, *CER8*, *CER10*, *LIPID TRANSFER PROTEIN1* (*LTP1*), *LTP2*, *WAX INDUCER1* (*WIN1*), etc. [13]. These wax-biosynthetic genes can be placed into three categories: the backbone genes, regulation genes, and transporters. For example, the backbone gene *CER1* interacts with *CER3* with both jointly responsible for the synthesis and modification of VLC-alkane [14]. *CER3* catalyzes the VLC-acyl-CoAs to VLC-acyl and then *CER1* transforms it to the VLC-alkanes [14]. In addition, both genes are also pleiotropic and involved in morphogenesis in the wax exine of pollen and adaxial epidermal cell wall [15, 16]. Furthermore, *CER7* inhibits the RNA repressor of *CER3* to positively affect the biosynthesis of VLC-alkanes [17]. Heterodimers of *CER5* and the ABC transporter G family member 11 (ABCG11, also called white-brown complex homolog protein 11, the WBC11) are involved in the exportation of wax components to apoplast [18]. The *cer5* mutant of *Arabidopsis* revealed a lower content of epicuticular wax than the wild type [19], evidence that the expression of *CER5* affects the phenotype of leaf epicuticular wax.

Geographic differentiation of epicuticular wax composition, e.g. the carbon number of alkane backbones of wax, is thought to be a consequence of local adaptation to environmental differences such as precipitation and temperature [20]. This implies that leaf epicuticular wax is a functional trait, defined as the morpho-physio-phenological character contributing to environmental adaptation [21]. Functional traits are usually interdependent [22–24] and covariation among functional traits may reflect the causality or coordination between functional traits [22] which are adopted for maximizing performance in a given environment [25]. For example, a positive correlation was found between trichome density and latex production, and between C:N ratio and leaf toughness in milkweeds (*Asclepias* spp.) [26]. The trade-off between defensive characters, such as epicuticular wax, and growth-related characters, such as carbon, nitrogen, photosynthetic parameters, etc., is suggested as another interdependent relationship between functional traits [27–29]. The allocation of resources in physical and chemical defense was hypothesized to reduce unnecessary waste of resources, as another trade-off strategy for adaptation [30–32], or alternatively, was suggested as an integrated defense strategy against a wide range of herbivores [33].

The objective of this study was to test the adaptive hypothesis of leaf epicuticular wax in *Lithocarpus* and determine the relationship between leaf abaxial epicuticular wax crystal (LAEWC) and other functional traits. Genus *Lithocarpus* is a group of tropical and subtropical Asian tree species commonly known as stone oaks [34, 35] grouped with *Castanopsis*, *Castanea*, *Quercus*, and *Chrysolepis* [36, 37]. All of the *Lithocarpus* species whose chromosome numbers have been checked are reported to be 2n = 24 and are unlikely to be polyploids ([38]; The Index of Plant Chromosome Numbers, http://www.tropicos.org/Project/IPCN). Roughly half the known species are locally endemic to mainland China, adjacent islands, and Borneo [39–41], suggesting the species of this genus are highly adaptable to environmental changes. The adaptive characteristic is revealed in phenotypes, such as differences in epicuticular wax crystals and trichomes on leaf surface [42, 43], and reflect environmental variations. Drastic climate change during middle-to-late Miocene to the Quaternary (Pliocene and Pleistocene) is suggested as a major force affecting the current distribution and diversification rate of *Lithocarpus* [44]. Past climate change attributed to glacial oscillations which had a major influence on temperature and precipitation, may have directly or indirectly affect plant growth due to limits on water usage and photosynthesis efficiency [45]. Here we use *Lithocarpus* as a way of answering the question of whether the trait variation of leaf epicuticular wax adaptively reflects environmental change with other co-varied ecophysiological traits.

After confirming the presence/absence of leaf epicuticular wax by microscopic observation, three questions were explored in this study: (1) Are LAEWC related genes adaptively evolved? (2) Is the presence/absence of LAEWC co-varied with ecophysiologically functional relevant traits (e.g. chemical defensive and photosynthetic characters)? (3) Is the presence/absence of LAEWC related to environmental variations? We also calculated the time when the positive selection of the trait took place as well as tested the phylogenetic signal (PS) of the ecophysiological traits and environmental factors with the species evolution and gene evolution for understanding the trends of co-evolution of these functional traits. This study integrates evidence from ecophysiological and climatic data with evolutionary genetic analyses to illustrate the adaptive evolution of LAEWC of *Lithocarpus*.

## Methods

### Plant materials

Fourteen *Lithocarpus* species grown in the Fushan Botanical Garden, a long-term ecological research (LTER) site for the subtropical forest in northern Taiwan, were collected for morphological, genetic, and ecophysiological surveys.

### Scanning electron microscopy

Scanning Electron Microscopy (SEM) was conducted to check for the presence/absence of the epicuticular wax crystals on the leaf surfaces using the tabletop SEM TM3000 (Hitachi, Tokyo, Japan). Small pieces of fresh blades (5 mm × 5 mm) between the leaf margin and midrib were cut for microscopic observation and at least two individuals were adopted for checking the presence/absence of the epicuticular wax crystals at an accelerating voltage of 15 kV in order to obtain high image resolution signals.

### Molecular techniques

We sampled fresh leaves from one individual of each species, the fresh leaves were stored in the RNAguardian solution (MBGEN Bioscience, Taipei, Taiwan) at − 80 °C. RNA extraction followed Gambino et al.’s [46] approach and removed sugars and polyphenols using CTAB lysis buffer and genomic DNA with TRIzol (Life Technologies Corp. California, USA). In order to understand genetic variation and to test selective pressure by comparing the evolutionary rates of genes, four candidate genes involved in wax backbone synthesis (*CER1* and *CER3*), regulation (*CER7*), and transportation (*CER5*) were chosen for cDNA sequencing. Other genes such as *WIN1*, *waxy*, and *ECR* which are also involved in wax synthesis failed to amplify and could not be used for this study. Therefore, only the four *CER* candidate genes were identified as LAEWC related genes in this study. Six wax-unrelated genes (*CAP*, *DGD*, *ESRK*, *FAD*, *SAHH*, and *SAM*) were also sequenced as reference genes from genomic DNA. All selected genes, including the four LAEWC related genes and six wax-unrelated genes, are homologous based on specific primer selection and multiple checks with by sequencing of the cloned amplified products. Primer sequences, sequence information including sequence length and percentage of coding region and GenBank accession numbers are listed in Additional files (Additional file 1: Table S1). These genes have functions in other tissues including pollen and adaxial epidermis in other plant species [15, 34] so we focus on those genes expressed in leaf tissue of species with and without LAEWCs. The amplified cDNA fragments were cloned using a yT&A cloning kit (Yeastern Biotech, Taipei, Taiwan). Three clones from each species were chosen for subsequently sequencing and the M13F/M13R were used as the primer for sequencing. Bidirectional DNA sequencing was conducted using ExoSAP-IT (Thermo Fisher Scientific Inc., Waltham, MA, USA) and ABI BigDye 3.1 Terminator Cycle Sequencing Kit (Applied Biosystem, Foster City, CA, USA) and conducted by ABI PRISMH® 3730XL DNA Sequencer (Perkin-Elmer, Foster City, CA, USA). Due to little variation in chromosome number in the *Lithocarpus* species (almost all reported species are 2n = 24, [38]; The Index of Plant Chromosome Numbers, http://www.tropicos.org/Project/IPCN) and the unlikelihood of polyploidy, the possibility that different clones are homoeologs was not considered in this study. All sequences presented in this study were deposited in the NCBI GenBank under Accessions KY458808-KY458955.

### Genetic analyses

DNA sequences were aligned and checked with the assistance of BioEdit [47]. The aligned sequences of every gene were used for phylogenetic reconstruction by the neighbor-joining (NJ) method, maximum-likelihood (ML) method, and Bayesian inference (BI) using MEGA 6.0 [48], PhyML 3.0 [49], and MrBayes 3.2 [50], respectively. For NJ tree reconstruction, the maximum composite likelihood model was used as the nucleotide substitution model and pairwise deletion was set for gap treatment. For PhyML, nearest neighbor interchange (NNI) was used for tree searching and approximate likelihood-ratio test (aLRT) for estimating branch support. For BI tree, two parallel Markov chain Monte Carlo (MCMC) simulations of 10 million generations with 10% burn-in were used for obtaining the consensus tree. Six reference genes were also used for reconstructing the species tree. Due to the unavailability of the sequences of closest outgroup *Chrysolepis*, we chose *Quercus robur*, *Castanea mollissima*, and *Fagus sylvatica* as outgroup in BEAST 1.8.2 [51]. One-hundred million years ago (Mya) as the origin time of Fagaceae [52] and 60 Mya when *Fagus* and *Castanea* diverged [52] were used as calibration points for molecular dating under the lognormal relaxed molecular model. Yule’s pure-birth speciation model was chosen as the speciation model for species tree reconstruction. One-thousand million MCMC generations with a sampling of every 10,000 generations were used in summary statistic. The character state of LAEWC was then mapped to the species tree for hypothesizing trait-shift events. It should be noted that while the outgroup *Q. robur* is coded as “absence” of LAEWC, most of *Quercus* species revealed “presence” [53]. The closest outgroup *Chrysolepis* reveals “absence” of LAEWC [54] but the sequences and materials of that outgroup were unavailable for this study, so we adopted *Q. robur* for rooting but kept the “absence” state in outgroup LAEWC.

**Fig. 1:**
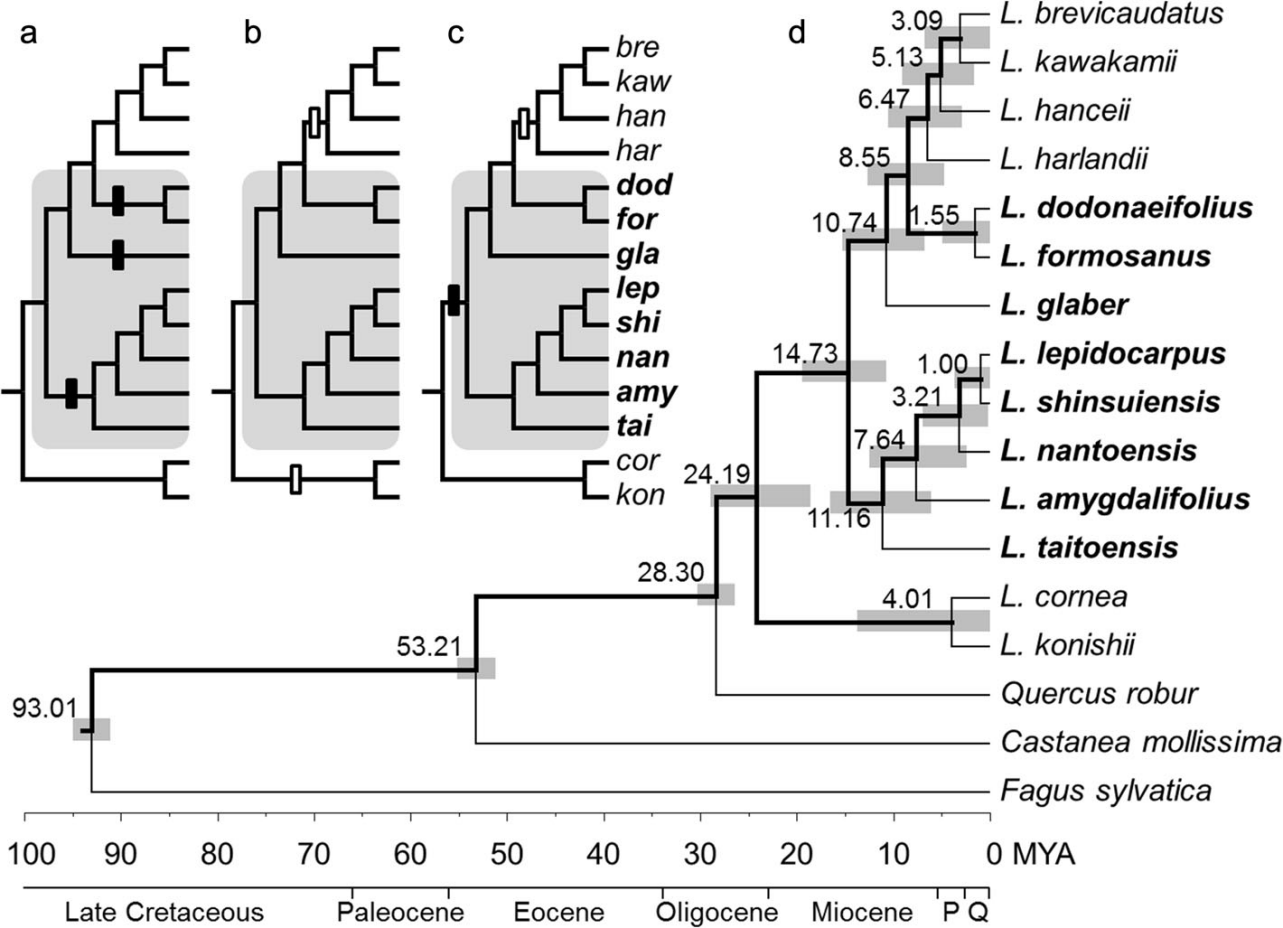
Species tree and hypotheses of positive selection on LAEWC trait-shift events. Three positive selection hypotheses are: (**a**) positive selection independently acted on lineages that gained LAEWC, (**b**) positive selection independently acted on lineages of lost LAEWC, and (**c**) positive selection acted on the ancestor of all LAEWC lineages and secondarily acted on the branch of loss LAEWC. The gray area indicates the species with LAEWC. The solid bars and hollow bars indicate the gain events and loss events of LAEWC, respectively. (**d**) The species tree of the studied *Lithocarpus* species reconstructed using six LAEWC unrelated genes. Bold branches indicate > 95% posterior probability supporting values for grouping. Species with LAEWC were marked in bold, which revealed non-monophyletic relationship of either LAEWC species or non-LAEWC species. Values near the nodes are the estimated splitting time (Mya) with 95% highest posterior density (gray bars). P and Q at the geological time scale axis are Pliocene and Quaternary, respectively

In order to test the selection signals of the four LAEWC related genes, substitution rates of all nucleotides of a gene (*K*), nonsynonymous nucleotide substitution rates (*Ka*) and synonymous substitution rates (*Ks*) of each LAEWC related gene were compared to that of reference genes. The dependent two-group Wilcoxon Signed Rank Test (WSRT) and simple linear regression (SLR) were used for testing the group differences between LAEWC related genes and reference genes. Due to the uncertainty of the ancestral state of LAEWC in *Lithocarpus*, we developed three scenarios which consider either the presence or absence of LAEWC*.* We then tested for the positive selection of trait shifts of LAEWC under three scenarios: (1) positive selection on independent gain events of LAEWC, (2) positive selection on independent loss events of LAEWC, and (3) positive selection on an early gain event of LAEWC and a following loss event (Fig. 1a-c). The branch model under the codeml analysis of PAML v.4 [55] was used for hypotheses testing and the *Ka*/*Ks* (*ω*) > 1 was taken as the signature of positive selection. The model of constant evolutionary rate constraint on *ω* < 1 around all branches of the tree was used as the null hypothesis (Fig. 1). Likelihood ratio test (LRT), which calculates the 2× differences of log likelihood between null and alternative hypotheses (2ΔL), was used for evaluating the best fit selective hypothesis by *χ*^2^ test.

### Ecophysiological measurements and analyses

The presence or absence of LAEWC was suggested to be related to photosynthetic efficiency and water use efficiency (WUE = net photosynthetic rate/transpiration rate) [56, 57], carbon storage efficiency [57], and insect and pathogenic stress defenses [58–60], etc. Therefore, several environmental and ecophysiological parameters were measured for correlation with the LAEWC trait. However, as the experiments were all confined to plants growing in the Fushan Botanical Gardens and not in their original habitats, not all environmental factors could be directly measured or taken into account. In addition, WUE related characters such as stomata morphology of this genus have been reported in previous studies not to vary significantly between species [42, 43]. First, we measured the phytochemical yield of photosystem II (YII), which is an indication of energy used in photochemistry by photosystem II under steady-state photosynthetic lighting conditions, i.e. as an index of photosynthetic efficiency. More than 20 samples per species (*n* = 20~ 52) were measured for YII and three measurements of every leaf sample were averaged. YII was measured using MINI-PAM-II Photosynthesis Yield Analyzer (Heinz Walz GmbH, Germany).

Phenolic acids (PA) are a group of secondary metabolites related to pigmentation, growth, reproduction, resistance to pathogens and herbivores, etc. and represent adaptive characters that have been subjected to natural selection [61]. The PA are also thought to serve a chemical ecological role in plant resistance to fungal pathogens and phytophagous insects [61]. Dried leaves (0.1 g) were placed in a 60% methanol solution (contain 3% HCl), and then subjected to centrifugation for 10 min at 4 °C (3000 rpm). Total phenolic content was determined with the Folin-Ciocalteu reagent according to a procedure described by Singleton and Rossi [62]. Briefly, 100 μL of the sample was mixed with 2 mL 2% (*w*/*v*) sodium carbonate solution for 2 min, and then 2 mL of 50% Folin-Ciocalteu reagent was added into the reaction mixture. The absorbance readings were taken at 750 nm after incubation at room temperature for 30 min. Gallic acid was used as a reference standard, and the results were expressed as milligram gallic acid equivalent (mg GAE)/g dry weight of herbal material. All species were measured four to five times and any outliers in the data discarded.

Carbon content (C) is attributed to the lignin, cellulose, and carbohydrates [63], and is positively associated with environmental light conditions and negatively correlated with insect herbivore abundance [64]. Leaf nitrogen content (N) is an integral component of protein RuBisCO and tends to correlate with the maximum photosynthetic rate [65] and is positively related to growth rates and energy supply [66]. The leaf C/N ratio is a proxy of nutrient limitation (cf. [67–69]) and usually correlates with the potential growth rate (cf. [69]). In addition to the leaf C and N content, we also measured the isotopic signature of leaf materials using the ratios of stable isotopes (R) of ^13^C/^12^C and ^15^N/^14^N and calculated the delta (δ) notation by the equation δ = [(*R*_samp_/*R*_std_)-1] × 1000‰, where the *R*_samp_ and *R*_std_ are the isotopic ratios of samples and international standards (Vienna Pee Dee Belemnite for δ^13^C and air N_2_ for δ^15^N), respectively. Since during the carbon fixation process of photosynthesis, the RuBisCO can more easily utilize the ^12^CO_2_ that may cause a lower concentration of ^13^C than the atmospheric ^13^C, the δ^13^C can accurately reflect the carbon fixation (CO_2_ uptake) efficiency of photosynthesis; similarly, the δ^15^N can also more accurately reflect the reaction efficiency of RuBisCO than leaf N content [70]. The δ^15^N is also suggested as an integrating parameter conditioning plant responsiveness (e.g., photosynthesis and water transpiration) to environments. For measuring the C, N, δ^13^C, and δ^15^N, leaf samples (*n* > 5 per species) were completely dried (oven-dried at 50 °C > 7 days) and ground into powders and mixed thoroughly. Dried powder samples were sent to SGS Taiwan Ltd. for measuring the C, N, δ^13^C, and δ^15^N following the protocol of Carter and Barwick [71].

Logistic regression was performed using the generalized linear model for testing the correlation of the ecophysiological traits with the presence/absence of LAEWC. LRT was used for comparing the null model. Phylogenetic principal component analysis (pPCA) [72] was conducted using all of the ecophysiological measurements and the altitudinal factors (the lowest (min Alt), highest (max Alt), middle altitudinal distributions (mid Alt), and the altitudinal range of distribution (ΔAlt)), with implementation of the R packages phytools [73]. We further tested whether these traits were synchronously co-adapted with the LAEWC related genes by PS tests. Pagel’s *λ* [74] and Blomberg’s *K* [75], which operate under the assumption of Brownian motion of trait evolution [76], were used for testing the significance of phylogenetic correlation with traits. These analyses were based on the species tree and four gene trees of the LAEWC related genes. If the ecophysiological trait revealed significant PS with the species tree, the trait was suggested to fit the phylogenetic niche conservatism hypothesis (PNC, if *K* > 1 [77]), or the character evolution fits a randomization process and is unrelated to the species evolution [77, 78]. If significant PS was detected with the gene tree, the trait was suggested to be coadapted with this gene.

## Results

### The SEM observation and differential LAEWC trait between species of *Lithocarpus*

**Fig. 2:**
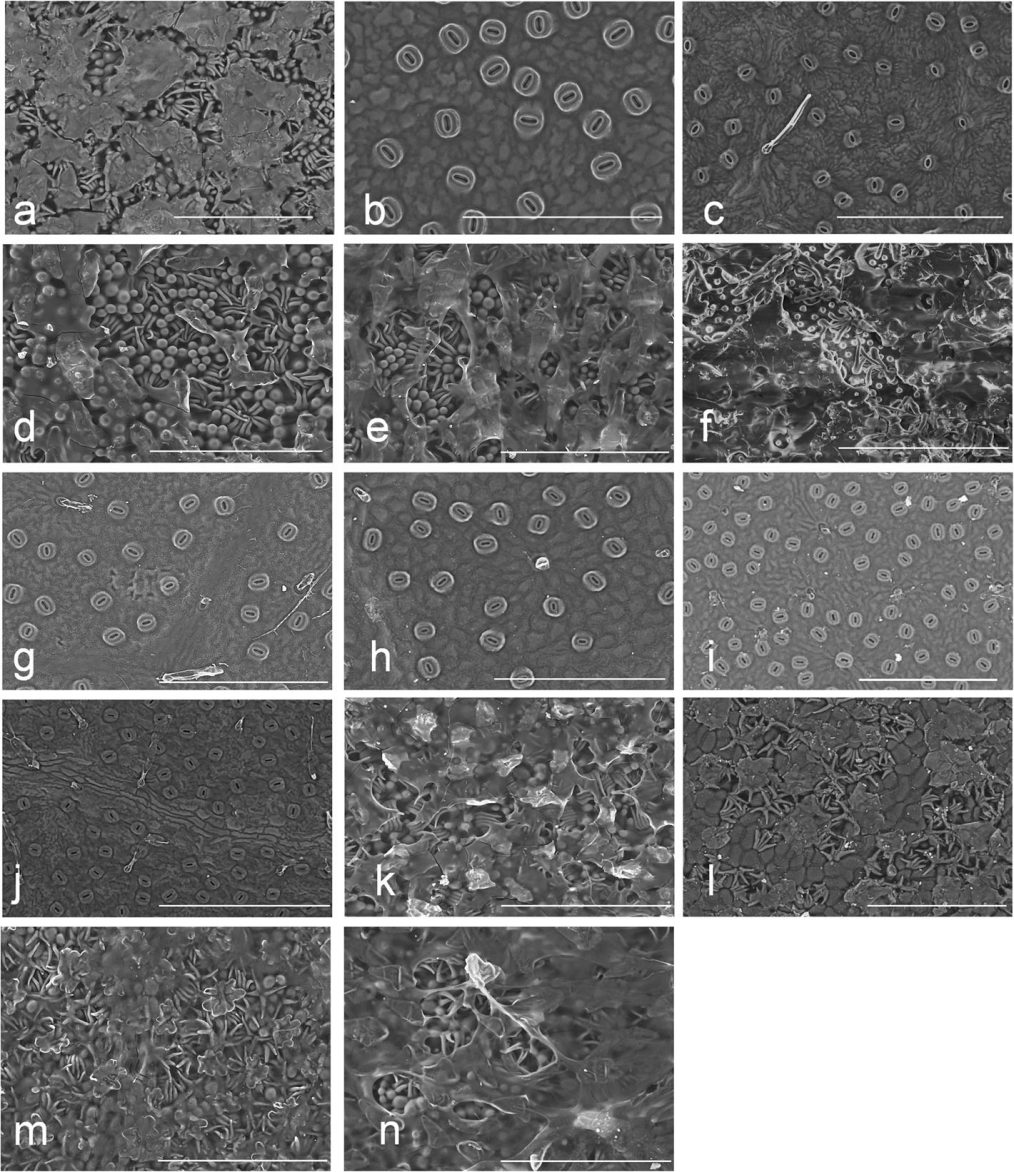
Details of the abaxial layer of leaf epidermis of species of the genus *Lithocarpus* using Scanning Electron Microscope (SEM). **a** *L. amygdalifolius*; (**b**) *L. brevicaudatus*; (**c**) *L. cornea*; (**d**) *L. dodonaeifolius*; (**e**) *L. formosanus*; (**f**) *L. glabe*r; (**g**) *L. hanceii*; (**h**) *L. harlandii*; (**i**) *L. kawakamii*; (**j**) *L. konishii*; (**k**) *L. lepidocarpus*; (**l**) *L. nantoensis*; (**m**) *L. shinsuiensis*; (**n**) *L. taitoensis*. The scale bar represents 200 μm

In all examined *Lithocarpus* species, the stomata are presented on the abaxial surface only with no infraspecies variations (Fig. 2). According to the SEM observation, eight species have LAEWC, including *L. amygdalifolius*, *L. dodonaeifolius*, *L. formosanus*, *L. glaber*, *L. lepidocarpus*, *L. nantoensis*, *L. shinsuiensis*, and *L. taitoensis* (Fig. 2). The LAEWC are mostly thin film and flaky (Fig. 2). The leaf abaxial trichomes were observed accompanying the presence of the LAEWC (Fig. 2). In contrast, the other six species (*L. brevicaudatus*, *L. cornea*, *L. hanceii*, *L. harlandii*, *L. kawakamii*, and *L. konishii*, Fig. 2) lack LAEWC and show very few and sparse trichomes, presenting a glabrous and stomata-naked surface. The adaxial surfaces of all six examined were glabrous and lacked epicuticular wax crystals (Additional file 1: Figure S1). To ensure that the presence/absence of LAEWC is not plasticity and environmental influences, we also check consistency between our results and specimens from previous study [42]. All species adopted in both our study and previous study show consistency in presence/absence in LAEWC, and revealed strong stability in presence/absence in LAEWC.

In general, five types of abaxial leaf surface were classified: (i) solitary unicellular trichomes growing through wax and stellate trichomes in the crack of the film-shaped wax crystals (Fig. 2a, d, m, n); (ii) almost full coverage of film-shaped wax crystals in the leaf abaxial surface (Fig. 2e); (iii) dense of solitary unicellular trichomes growing through pieces of wax crystals (Fig. 2f); (iv) solitary unicellular trichomes growing through pieces of wax films with uncovered stellate trichomes (Fig. 2k, l); (v) the naked stomata and no wax crystals covering the leaf abaxial surface (Fig. 2b, c, g, h, i, j). In addition, leaf abaxial trichomes that coexist with the LAEWC vary between species: the parallel tuft trichome (Fig. 2d), stellate trichomes with papillae (Fig. 2a, d, e, k, n), solitary unicellular trichome (Fig. 2f), and broad base trichomes (Fig. 2c and j). Appressed parallel tufts were also present in eight species with LAEWC (Fig. 2a, d, e, f, k, n). Some species are glabrous on the leaf abaxial surface (Fig. 2b, c, g, h, i, j). The pattern of anticlinal walls and the shape of the epidermal cells are the main characteristics of the adaxial epidermis. Anticlinal walls are curved, straight, or sinuate. Epidermal cells are irregularly rounded, regular, or polygonal. No stomata were found on the adaxial surface (Additional file 1: Figure S1). The variety of trichomes between species are not the topic of this study but merit further investigation.

### The character state of LAEWC is not related to the spatial distribution

According to species distribution modeling conducted by Maxent [79], with implementation of the R packages dismo [80], several species have overlapping distributions and these distributions are not obviously different between species with LAEWC and without LAEWC (Additional file 1: Figure S2). This result suggests the presence/absence of LAEWC is not related to the current distribution of species or may not reflect fine-scale environmental differences within a small island.

### Phylogenetic analyses does not support a single trait-shift event of LAEWC

The species tree revealed that species with LAEWC do not form a single clade; nor do species without LAEWC (Fig. 1d). A species tree reconstructed with six reference genes showed an ancestry for the studied *Lithocarpus* species dating to 24.19 Mya (Fig. 1d). Three independent lineages (clades) of species with LAEWC could be coalesced since 11.16 Mya, 10.74 Mya, and 1.55 Mya, and branches of non-LAEWC species could be coalesced to 6.47 Mya and 4.01 Mya (Fig. 1d). These dates are roughly in line with the late Miocene and Pliocene, which is older than the formation of Taiwan Island (less than 4 Mya) where the studied species are distributed [81–83]. Such dating indicates that the occurrence of these species in Taiwan was not a consequence of radiation after colonizing Taiwan Island but could point to multiple colonizing events. This inference was also supported by the chloroplast DNA tree with more taxa (Additional file 1: Figure S3). This result implies that the presence/absence of LAEWC was not the derived character for adapting to the island environment but could be a relict of an adaptive trait. The ancestor state (presence or absence) of LAEWC of *Lithocarpus* remains unknown because the phylogenetically closed genera (outgroups, *Quercus* and *Castanea*) have varied character states in LAEWC and ambiguous ancestral states inferred by MCMC simulation under the equal-rate (ER) model according to the chloroplast DNA tree (Additional file 1: Figure S3). Nevertheless, the phylogenetic analysis still provides evidence that the presence or absence of LAEWC is not a single event in *Lithocarpus* evolution (Fig. 1 and Additional file 1: Figure S3). Therefore, at least three evolutionary scenarios of gain or loss of LAEWC could be hypothesized: (a) gaining LAEWC are independent events, (b) losing LAEWC are independent events, and (c) losing LAEWC is a reversal event (Fig. 1a-c).

### Incongruent tree topologies between gene trees and species tree inferred from reference genes

**Fig. 3:**
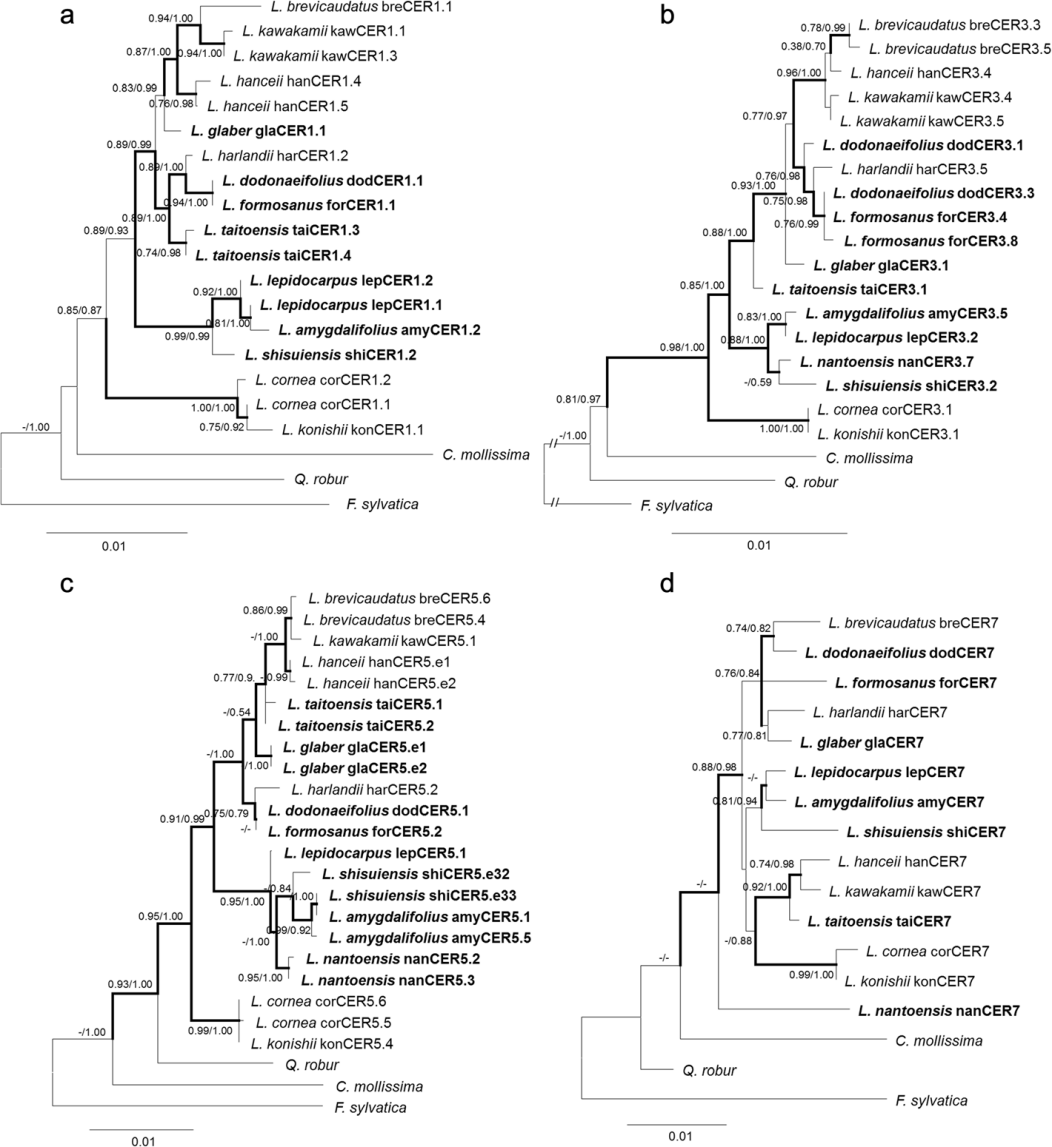
Gene trees of LAEWC related genes (**a**) *CER1*, (**b**) *CER3*, (**c**) *CER5*, and (**d**) *CER7*. Tree topologies shown here are based on the neighbor-joining method and the branches with bold indicate bootstrap values > 50% for supporting the deriving groups. Values of the nodes indicate the posterior probabilities of the supporting values inferred by the maximum likelihood (ML) and Bayesian inference (BI) methods (ML/BI). Dashes indicate the posterior probability < 50%. The operational taxonomic units labeled in bold are the species with LAEWC. Codes after the species name are the haplotypes cloned in this study

Generally, the grouping pattern of species with LAEWC and without LAEWC in the gene trees of *CER1*, *CER3*, and *CER5* are similar to that of the species tree reconstructed from six reference genes, except that the haplotypes of *L. harilandii* (species without LAEWC) was grouped with *L. dodonaeifolius* (with LAEWC) (Fig. 3a-c). Certain species possess two haplotypes of the wax related genes and two haplotypes do not form a single clade, which was likely to be caused by gene duplication or retention of ancestral polymorphism [84]. According to previously reported in model species, only one copy can be found in all of the *CER* candidate genes from all model species [10, 14, 85, 86]. Besides, most species do not possess more than one haplotype. Therefore, different haplotypes within the species were regarded as intraspecific polymorphism rather than different members of a small gene family caused by duplication. In addition, within the LAEWC group, which did not form a monophyly in either gene tree or species tree, the *L. taitoensis* grouped with *L. dodonaeifolius*, *L. formosanus*, and *L. glaber* in gene trees of *CER1*, *CER3*, and *CER5* (Fig. 3a-c), but grouped with *L. lepidocarpus*, *L. nantoensis*, *L. shinsuiensis*, and *L. amygdalifolius* in the species tree (Fig. 1). The gene tree of *CER7* is different from the other gene trees and the species tree, which revealed a mosaic topological distribution of LAEWC species with non-LAEWC species (Fig. 3d). Furthermore, all clones of *CER7* are monomorphic within species (one haplotype per species), revealing strong genetic constraint of species.

### Comparison of evolutionary rates of LAEWC related and unrelated genes

The null hypothesis of WSRT is that there is no difference in the median between pairs of observations, while the null hypothesis of SLR implies independent distribution (no relationships) between two sets of observations. Hence, we use WSRT to test the difference in evolutionary rates between LAEWC related and unrelated genes and the rate of functional and unfunctional change of LAEWC related genes. We also conducted the SLR to test whether the LAEWC related genes evolve independently of species divergence or not. The Wilcoxon test shows significant differences in *K* between LAEWC unrelated (reference) genes and *CER3* (*P* < 2.2 × 10^− 16^), *CER5* (*P* = 1.13 × 10^− 6^), and *CER7* (*P* = 0.0005), but non-significant difference between reference genes and *CER1* (*P* = 0.734) (Additional file 1: Figure S4A-D), which indicates that the evolutionary rate of *CER1* does not differ from that of reference genes. Since most regions of the reference genes are non-coding regions (introns), which were suggested to be free of selection, we compared the *K* of reference genes with the nonsynonymous substitution rate (*Ks*) of LAEWC related genes. Significant WSRT results (*P* < 0.05 in all comparisons) suggested different rates between the LAEWC related genes and reference genes, but the positive significance of the SLR suggests that the silent mutations of these LAEWC related genes still follow the sequence of species divergence (Additional file 1: Figure S4E-H). When comparing the *Ks* of LAEWC related genes to the nonsynonymous substitution rate (*Ka*), very significant lower *Ka* for these LAEWC related genes than *Ks* was found by WSRT (Additional file 1: Figure S4I-L). Taken together with the comparisons of *Ks* of LAEWC related genes and the *K* of reference genes and the *Ka* vs. *Ks* of the LAEWC related genes, we concluded that these LAEWC related genes evolve more rapidly than other wax unrelated genes while experiencing strong selective pressure to constrain amino acid changes.

### Hypothesis testing indicates positive selection of *CER1* on the gain events of LAEWC

The results show that, except in the *CER1* scenario of gaining LAEWC (hypothesis 1, Fig. 1a; 2ΔL = 6.754, *P* = 0.009, Table 1), the constant-rate model (M0) cannot be rejected in other LAEWC-related genes under all evolutionary scenarios of trait-shift events. Although three branches that inferred gaining LAEWC have an estimate of ω > 1 in *CER1* under hypothesis 1, not all branches were detected as having the same amino-acid replacement: only one and two amino acid replacements were found in *L. glaber* and in the branch of the common ancestor of *L. dodonaeifolius* and *L. formosanus*, respectively. No replacement was found in the common ancestor of other LAEWC species, implying a false positive. The gain of LAEWC in the early stages of the evolution of *Lithocarpus* was suggested to take place at multiple times and independently from a probable ancestral state of absence of LAEWC (Additional file 1: Figure S3). However, certain lineages of LAEWC loss were inferred (Additional file 1: Figure S3), but none of these “loss” events were suggested to be under selective pressure (Table 1). We also tested whether positive selection acts on retaining LAEWC by allowing ω > 1 in all derived lineages after gaining or losing the LAEWC trait. The results show that the null hypothesis of constant-rate model cannot be rejected in all situations (Additional file 1: Table S2). This implies that the episodic gains of LAEWC may be responsible for adaptive radiation rather than lasting divergence.

**Table 1:** Results of likelihood ratio test of hypotheses of positive selection on LAEWC trait shift. The result shows that most LAEWC related genes were not positively selected during the trait shift of LAEWC except the *CER1* under the hypothesis 1 (positive selection independently acted on the gain of LAEWC)

Hypothesis^a^	Gene	ln*L*_*0*_^b^	ln*L*_*A*_^b^	2Δ*L*	df	*P*
Hypothesis 1	*CER1*	− 2969.327	− 2965.950	6.754	1	0.009
	*CER3*	− 2923.209	−2923.041	0.338	1	0.561
	*CER5*	− 3516.913	−3516.577	0.670	1	0.413
	*CER7*	−2009.028	−2008.247	1.561	1	0.211
Hypothesis 2	*CER1*	−2969.327	−2969.322	0.010	1	0.919
	*CER3*	−2923.209	−2923.133	0.153	1	0.696
	*CER5*	−3516.913	−3516.908	0.010	1	0.921
	*CER7*	−2009.028	−2007.660	2.735	1	0.098
Hypothesis 3	*CER1*	−2969.327	− 2967.980	2.694	2	0.260
	*CER3*	−2923.209	−2923.210	0	2	NA
	*CER5*	−3516.913	−3516.913	0	2	1
	*CER7*	−2009.028	−2007.662	2.731	2	0.255

^a^Hypothesis 1: Fig. 1a; Hypothesis 2: Fig. 1b; Hypothesis 3: Fig. 1c

^b^ln*L*_*0*_: natural logarithm of the likelihood of null model; ln*L*_*A*_: natural logarithm of the likelihood of alternative model

### Association of PA with the evolution of LAEWC-related genes

**Fig. 4:**
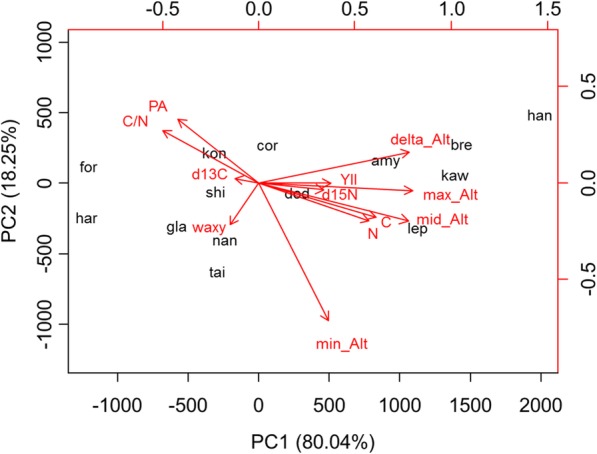
Phylogenetic principal component analysis (pPCA) conducted with the reference tree. Black wording indicates abbreviation of each species (see Fig. 1), while the red wording indicates ecophysiological traits. (C:carbon content; N: nitrogen content; PA: phenolic acid; C/N: ratio of carbon and nitrogen content; d13C: δ^13^C; d15N: δ^15^N; waxy: LAEWC state; Alt: altitude; YII: phytochemical yield of photosystem II)

To understand whether the gain of LAEWC benefits the adaptation of trees, certain ecophysiological traits concerning photosynthesis or stress defense, etc. were measured (Additional file 1: Table S3) and used as independent variables to correlate with the character of LAEWC. Except the C and C/N ratio (2ΔL = 5.791 and 3.845, *P* = 0.016 and 0.050, respectively, Table 2), the empty model cannot be rejected by the models with other independent variables (Table 2). Even so, the effect of C and C/N still fails to significantly predict the character state of LAEWC (*P* = 0.111 and 0.090, respectively, Table 2). These results suggest that the presence/absence of LAEWC does not covary with current ecophysiological traits, i.e. is unrelated to current environmental pressures. This result is consistent with the lack of covariance of LAEWC with other ecophysiological characters in phylogenetic principal component analysis (Fig. 4).

**Table 2:** Logistic regression of every ecophysiological trait with the presence or absence of LAEWC, which revealed non-significant correlation with LAEWC in all ecophysiological traits

Variable	Estimate	SE	z value	Pr (>|z|)	log L	LRT^b^
Null^a^	–	–	–	–	− 9.561	–
YII	14.740	14.780	0.997	0.319	−9.018	0.297
PA	0.033	0.031	1.045	0.296	−8.895	0.249
δ^13^C	0.665	0.748	0.889	0.374	−9.130	0.354
δ^15^N	−1.412	1.072	−1.317	0.188	−8.174	0.096
C	94.100	58.980	1.595	0.111	−6.665	0.016
N	− 176.590	126.725	−1.393	0.163	−8.348	0.119
C/N	0.171	0.101	1.697	0.090	−7.638	0.050

^a^the empty model

^b^the likelihood ratio test in which the model is compared with the empty model using 2× delta log likelihood (2ΔL)

PS analyses showed that no ecophysiological variables reflect species evolution, but the PA can reflect the gene evolution of *CER1* (*K* = 0.483, *P* = 0.014) and *CER3* (λ = 0.870, *P* = 0.011; *K* = 0.549, *P* = 0.007, Table 3). Significant PS of PA in the *CER1* and *CER3* gene trees suggest coevolution of the PA and LAEWC backbone genes. However, the hypothesis of phylogenetic conservatism in both chemical (PA) and physical (LAEWC) stress defense is rejected due to the *K* < 1. In addition to the PA, the C/N ratio that represents the potential growth rate and nutrient limitation may also reflect the evolution of *CER5* gene (Blomberg’s *K* = 0.290, *P* = 0.034, Table 3). However, nonsignificant PS detected in C, N, δ^13^C, and δ^15^N, and YII in the *CER5* gene tree and other gene trees (Table 3) indicate no relationship between the adaptive evolution of LAEWC and photosynthesis efficiency, water usage efficiency, and/or nutrient allocations.

**Table 3:** Phylogenetic signals of ecophysiological traits with species tree and four LAEWC-related-gene trees

	Test	YII	PA	δ^13^C	δ^15^N	C	N	C/N
Species tree	Pagel’s λ	6.90E-05	0.637	6.90E-05	0.363	6.90E-05	6.90E-05	6.90E-05
	Blomberg’s K	0.267	0.326	0.445	0.458	0.241	0.170	0.184
CER1 gene tree	Pagel’s λ	0.567	0.640	6.70E-05	0.527	4.25E-05	6.70E-05	7.55E-05
	Blomberg’s K	0.164	0.483*	0.353	0.355	0.096	0.150	0.141
CER3 gene tree	Pagel’s λ	6.79E-05	0.870*	6.79E-05	0.612	6.79E-05	6.79E-05	6.79E-05
	Blomberg’s K	0.201	0.549*	0.205	0.254	0.161	0.173	0.204
CER5 gene tree	Pagel’s λ	6.72E-05	0.453	0.077	0.519	6.72E-05	6.72E-05	6.72E-05
	Blomberg’s K	0.211	0.088	0.206	0.196	0.074	0.209	0.290*
CER7 gene tree	Pagel’s λ	6.90E-05	0.637	6.90E-05	0.363	6.90E-05	6.90E-05	6.90E-05
	Blomberg’s K	0.267	0.326	0.445	0.458	0.241	0.170	0.184

*P < 0.05

## Discussion

### Synchronous character states in the LAEWC and trichomes

This study used 14 *Lithocarpus* species distributed on Taiwan Island to investigate the adaptive character of LAEWC by means of genetic and ecophysiological assessments. First, the SEM showed that eight of 14 species have an obvious film-shaped wax crystal structure on the leaf surface abaxial layer (Fig. 2) but only very sparse, or sometimes no, wax films were found on the leaf adaxial layer (Additional file 1: Figure S1). Lack of leaf adaxial wax crystals suggest no selective pressure or pressure of light intensity on the evolutionary shaping of leaf epicuticular wax in *Lithocarpus* (cf. [87]). In contrast, the differences in film-shaped wax crystals on the abaxial layer, where the stomata are mainly distributed, suggest the ecophysiological relevance of LAEWC in photosynthesis efficiency and/or water conservation. A study of grasses (*Agrosti stolonifera*, Poaceae) further indicated that the difference in distribution of the extracellular wax on leaf surfaces can also be related to the retention of salt at different localities [88]. Interestingly, the presence of trichomes was associated with the presence of LAEWC in our study materials (Fig. 2) and the diverse forms of trichome have also been reported and discussed in previous study [42, 43], although it should be noted that few exceptions have been reported in species which were not adopted in our study (e.g, *L. calophyllus* and *L. oleifolius* in [42]). Co-occurrence of these traits may suggest, for example, that synergistic functions for adaptation play the role of physical barrier in the defense against insect biting [87] or water repellency [89]. However, the trichome types are more complicated than the wax films in *Lithocarpus* (Fig. 2). Hence the trichomes’ ecophysiological functions, their potential linkage with wax films, the linkage of trichome morphogenesis related gene such as *MIXTA*-like [90], and adaptive characters are not discussed here.

### Functional independency of LAEWC to all ecophysiological traits

Most of the factors we measured do not reveal significant PS of the species tree, including photosynthesis efficiency, water usage efficiency, and/or nutrient allocation dependent characters. This is consistent with other leaf abaxial characters - for example, there are no distinguishable differences in morphology/distribution of stomata, abaxial epidermal cell walls, and epidermal hairs [42, 53] - although we could not exclude other factors driving adaptation in *Lithocarpus*. Among these factors, the contents of PA revealed significant PS with the gene trees of the backbone genes of the wax biosynthesis pathway (i.e. *CER1* and *CER3*, Table 3). The makeup of defensive chemicals usually directly reflects the herbivore stress of plants and reveals strong signs of positive selection [91, 92]. Cuticular or epicuticular waxes of the leaf surface are suggested to play a role in physical defense [60, 87]. Two hypotheses could explain the coevolutionary relationship between the chemical and physical defensive strategies: (1) the trade-off between the chemical and physical defensive investment that causes negative results of correlation [30–32], and (2) synergistic defense hypothesis suggests a positive correlation between chemical and physical trait values [33]. Similarly, the trade-off between the defensive and growth investments in plants could result in negative correlations between the defensive and photosynthesis (or nutrient) trait values. However, our study shows neither a positive nor a negative correlation between chemical and physical defensive traits, as well as no correlation between LAEWC and photosynthesis or nutrient uptake/storage indices (Table 2). This means that the trade-off hypothesis for the resource investment of chemical and physical defensive strategies is not supported. Our result is similar to that found in myrmecophilic plant *Macaranga tanarius* which showed that the chemical composition of the leaf revealed no correlation with the wax contents of the leaf surfaces [93]. However, in that case, a strong positive correlation with the density of trichomes was detected, suggesting complicated synergistic defensive strategies of plants [93]. This study echoes the above and considers trichomes worthy of future exploration. At the same time, the phylogenetic PCA revealed the opposite explanatory dimensions on the chemical defensive trait (PA) and other nutrient and photosynthesis indices (Fig. 4), showing support for the trade-off hypothesis between defensive and growth investment in *Lithocarpus*. Evidence in support of another hypothesis, that spreading the risks of resource investment resulted in general phenomenon of less or no association between defensive traits, i.e. independently evolving, was not uncovered [33].

### Evolutionary change of LAEWC co-adapts with the PA

Though we failed to find correlations between any ecophysiological trait and LAEWC (Table 2), our study has found evidence for a strong relationship between PA content and the evolution of backbone genes of the wax biosynthesis pathway. One of the backbone genes, *CER1*, was also found to be positively associated with gaining of LAEWC (Table 1), suggesting a synchronization of the relationship between the evolution of chemical and physical defensive traits. Although other potential functional interpretations could be considered in *CER1* - for example, pollen morphogenesis [15] and adaxial wax cuticle [16] - the possibility of adaptive interpretation in pollen or adaxial wax cuticle seems to be unlikely due to very little variation in pollen [94] and adaxial cuticle morphology [53, 54]. Therefore, we focus our discussion on defensive traits with LAEWC and *CER1*. Contrasting hypotheses of parallel evolution and coadaptation explain how the chemical (e.g. PA) and physical (e.g. LAEWC) defensive characters covary: the former describes synergistic evolution of both traits following species divergence, while the latter suggests synchronous changes of characters in response to the same stress but independent from the evolution sequence of the species. In this case, nonsignificant PS of the PA with the species tree has excluded the possibility of parallel evolution of these two traits. Several studies have shown independent or unlinked relationships between the chemical and physical defensive traits and do not support the parallel evolution hypothesis of two defensive strategies [42, 87, 93].

In contrast, a coadaptive evolutionary relationship in both PA and LAEWC was suggested due to significant PS of PA on the backbone genes of wax biosynthesis (Table 3). In addition, the positive selection on the *CER1* gene at the time of gaining LAEWC implies that the association of PA with the evolution of wax backbone genes reflects the adaptive consequence of character innovation. Besides *CER1*, none of the examined genes have signs of positive selection in any possible evolutionary scenarios (Table 1). Positive selection on backbone gene *CER1* suggests that the trait shift of LAEWC is the overriding functional change for adaption. Overexpression of *CER1*induced by osmotic stresses will increase the production and accumulation of the raw materials of cuticle wax, which was shown to increase plant susceptibility to bacterial and fungal pathogens as well as reduce cuticle permeability and soil-water deficit susceptibility in *Arabidopsis* [10].

Although the state of LAEWC in the common ancestor of the extant *Lithocarpus* is ambiguous, the gaining of LAEWC likely happened in the early stages of species divergence (Additional file 1: Figure S3). This idea is supported by the absence of LAEWC in the sister group, *Chrysolepis*, after observation by SEM [54]. The positive selection detected upon the of gaining of LAEWC indicates adaptive advantages of LAEWC in *Lithocarpus*. Furthermore, the selective pressure on *CER1* was not retained for maintaining the character (Additional file 1: Table S2). Functional traits of leaves such as the specific leaf area and hydraulic traits were suggested to be associated with the species niche and strongly reflect species distribution [95, 96]. However, modeling for species distribution based on current climate variables indicated no obvious differences between the species with and without LAEWC (Additional file 1: Figure S2). This may suggest that the trait of LAEWC may reflect past environmental change which led to trait shifts rather than current environmental realities.

### Late-Miocene-to-Pliocene climate change explains the trait-shift of LAEWC in *Lithocarpus*

The timing of the trait transition of LAEWC was suggested as the late Miocene and Pliocene (Fig. 1). Late Miocene to Quaternary climate change may coincide with the trait shift events of LAEWC in *Lithocarpus*. The Miocene climate was slightly warmer and wetter and spawned the monsoon system which has affected Asian vegetation [97–99]. After the late Miocene, the climate gradually cooled and became arid [100, 101]. The late-Miocene-to-Pliocene climate change accelerated the diversification of insular species [102–105], which may be partly due to sea-level fluctuations accompanying disjunction and connection between continents and islands [106]. The synergy of genetic isolation and adaptation to the rugged topography may have accelerated the speciation process in southeastern Asian islands.

Trait innovation has been found to enhance the diversification of several plants since the late-Miocene-to-Pliocene boundary. For example, the appearance of winged seeds in *Parrya* and *Diptychocarpus* of Brassicaceae may aid in plant colonization [107]. As with *Parrya* and *Diptychocarpus*, acquiring the LAEWC to increase survival by defending against insects may have accelerated species diversification in these tropical and subtropical stone oaks in the late-Miocene-to-Pliocene period at the same time that insect species were diversifying, including tropical-forest beetles [108], most genera of Nymphalid [109], and fruit fly (*Rhagoletis*, [110]). In addition, some plant species developed higher drought adaptability usually characterized by water-conserving traits (i.e. high hydraulic safety margins) and, as with the presence of LAEWC in *Lithocarpus*, show a higher potential of resistance to climate change [95]. However, although these traits can reflect environmental and climate change, they may act somewhat independently of one another [14], hence these traits were poorly correlated with each other (Table 2) in our study. This may be because the specific combination of traits may maximize species performance only in specific environments [111].

## Conclusions

The late-Miocene-to-Pliocene positive selection on backbone gene *CER1* of leaf epicuticular wax accompanying the content of PA of chemical defensive traits suggests the adaptive change and diversification rate coincides with the diversification of many herbivorous insects and potential link between PA, wax, and defensive adaptation in *Lithocarpus*. The selective pressure which produced this linkage did not persist over time; however, the trait transition has remained. None correlation of the wax trait with the other ecophysiological characters suggests functional independency. The evolutionary association in chemical and physical defensive strategies was suggested as a coadaptive phenomenon to resist multiple or complicated phytophagous stress instead of reflecting the parallel evolution of traits. Our study began with a morphological observation and concluded having found evidence of the synchronous adaptive change of defensive traits under past selective pressure, uncovered through genetic and ecophysiological analyses. We contribute a possible explanation of the mechanism for the diversification of tropical and subtropical forest species in the face of climate change.

## Additional file

Additional file 1: **Figure S1.** Detail of adaxial layer of leaf epidermis of the genus *Lithocarpus* using Scanning Electron Microscope (SEM). (A) *L. amygdalifolius*; (B) *L. brevicaudatus*; (C) *L. cornea*; (D) *L. dodonaeifolius*; (E) *L. formosanus*; (F) *L. glabe*r; (G) *L. hanceii*; (H) *L. harlandii*; (I) *L. kawakamii*; (J) *L. konishii*; (K) *L. lepidocarpus*; (L) *L. nantoensis*; (M) *L. shinsuiensis*; (N) *L. taitoensis*x. The SEM shows that no epicuticular wax crystals covered the leaf adaxial leaf surface in *Lithocarpus*. The scale bar represents 200 μm. **Figure S2.** The spatial distribution reconstructed according to the current sampling records from GBIF and predicted using a machine-learning algorithm, maximum entropy algorithms, implemented in Maxent [79]. **Figure S3.** Ancestral state inference of discrete characters using the Maximum likelihood framework assuming one-parameter equal rates (ER) of character transition model and summarizing the 300 simulated character reconstructions. **Figure S4.** Pairwise comparison of substitution rates (*K*) of LAEWC related genes and reference genes using simple linear regression (SLR) and dependent two-group Wilcoxon Signed Rank Test (WSRT). **Table S1.** Primer list and annealing temperatures used in this study. **Table S2.** Results of likelihood ratio test of hypotheses of positive selection on retaining the gain or loss of LAEWC trait. **Table S3.** Ecophysiological measurements and altitudinal distribution of *Lithocarpus* species tested in this study. (PDF 1094 kb)

## Abbreviations

2ΔL: 2× differences of log likelihoods between two models
ABC: ATP-binding cassette
ABCG: ABC transporter G family
aLRT: Approximate likelihood-ratio test
Alt: Altitude
BI: Bayesian inference
C: Carbon content
CER: ECERIFERUM
ER: Eequal rates
GAE: Gallic acid equivalent
*K*: Substitution rate of all nucleotides
*Ka*: Nonsynonymous substitution rate
*Ks*: Synonymous substitution rate
LAEWC: Leaf abaxial epicuticular wax crystals
LTER: Long-term ecological research
LTP: LIPID TRANSFER PROTEIN
M0: Constant-rate model
MCMC: Markov chain Monte Carlo
ML: Maximum-likelihood
Mya: Million years ago
N: Leaf nitrogen content
NJ: Neighbor-joining
NNI: Nearest neighbor interchange
PA: Phenolic acids
PNC: Phylogenetic niche conservatism
pPCA: Phylogenetic principal component analysis
PS: Phylogenetic signal
R: Stable isotope ratio
SEM: Scanning Electron Microscopy
SLR: Simple linear regression
VLCFAs: Very-long-chain-fatty-acids
WBC: White-brown complex homolog protein
WIN1: WAX INDUCER1
WSRT: Wilcoxon Signed Rank Test
WUE: Water use efficiency
YII: Phytochemical yield of photosystem II
ΔAlt: Altitudinal range of distribution
*ω*: *Ka*/*Ks*
