# Supplementary material for: Molecular genetic and biochemical evidence for adaptive evolution of leaf abaxial epicuticular wax crystals in the genus Lithocarpus (Fagaceae)
Source: BMC Plant Biol. 2018 Sep 17;18:196. doi: 10.1186/s12870-018-1420-4 (PMC6142356; doi:10.1186/s12870-018-1420-4)
Supplement: Supplementary file 1 — Figure S1. Detail of adaxial layer of leaf epidermis of the genus Lithocarpus using Scanning Electron Microscope (SEM). (A) L. amygdalifolius; (B) L. brevicaudatus; (C) L. cornea; (D) L. dodonaeifolius; (E) L. formosanus; (F) L. glaber; (G) L. hanceii; (H) L. harlandii; (I) L. kawakamii; (J) L. konishii; (K) L. lepidocarpus; (L) L. nantoensis; (M) L. shinsuiensis; (N) L. taitoensisx. The SEM shows that no epicuticular wax crystals covered the leaf adaxial leaf surface in Lithocarpus. The scale bar represents 200 μm. Figure S2. The spatial distribution reconstructed according to the current sampling records from GBIF and predicted using a machine-learning algorithm, maximum entropy algorithms, implemented in Maxent [79]. Figure S3. Ancestral state inference of discrete characters using the Maximum likelihood framework assuming one-parameter equal rates (ER) of character transition model and summarizing the 300 simulated character reconstructions. Figure S4. Pairwise comparison of substitution rates (K) of LAEWC related genes and reference genes using simple linear regression (SLR) and dependent two-group Wilcoxon Signed Rank Test (WSRT). Table S1. Primer list and annealing temperatures used in this study. Table S2. Results of likelihood ratio test of hypotheses of positive selection on retaining the gain or loss of LAEWC trait. Table S3. Ecophysiological measurements and altitudinal distribution of Lithocarpus species tested in this study. (PDF 1094 kb) [file 12870_2018_1420_MOESM1_ESM.pdf]

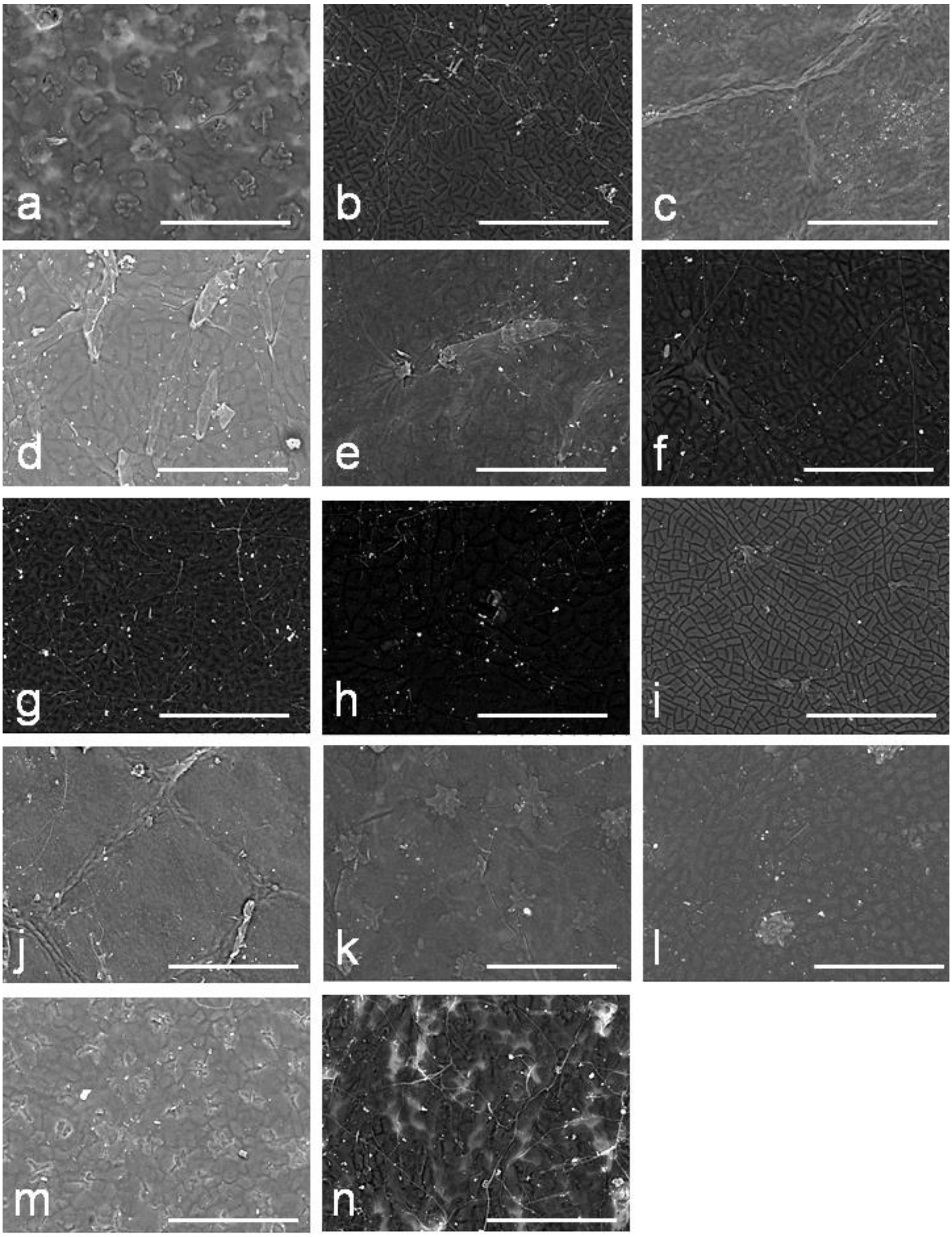

Figure S1. Detail of adaxial layer of leaf epidermis of the genus *Lithocarpus* using scanning electron microscope (SEM). (A) *L. amygdalifolius*; (B) *L. brevicaudatus*; (C) *L. cornea*; (D) *L. dodonaeifolius*; (E) *L. formosanus*; (F) *L. glabe*r; (G) *L. hanceii*; (H) *L. harlandii*; (I) *L. kawakamii*; (J) *L. konishii*; (K) *L. lepidocarpus*; (L) *L. nantoensis*; (M) *L. shinsuiensis*; (N) *L. taitoensis*x. The SEM shows no epicuticular wax crystals covered in the leaf adaxial surface in *Lithocarpus*.

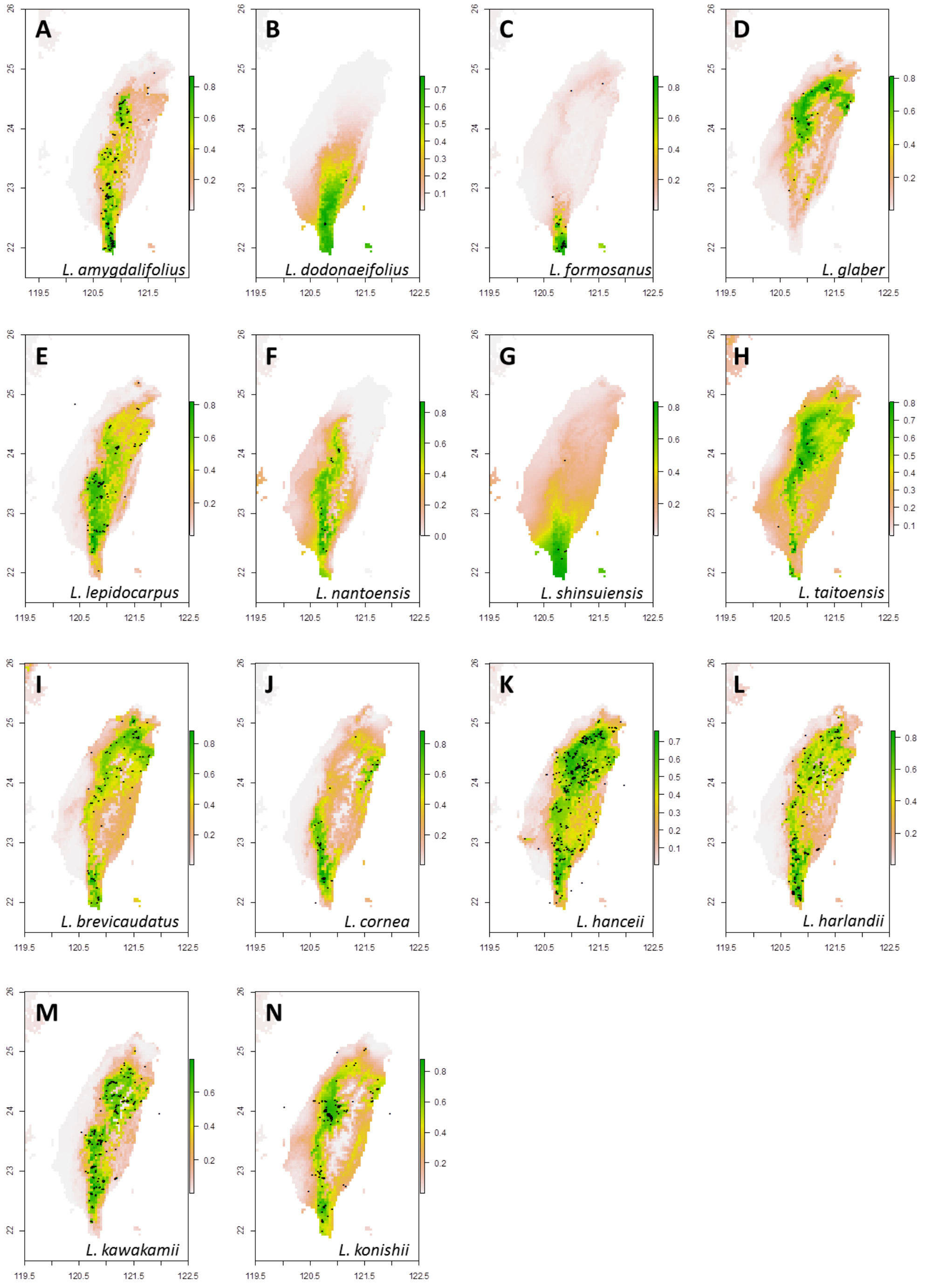

Figure S2. The spatial distribution reconstructed according to the current sampling records from GBIF and predicted using a machine-learning algorithms, maximum entropy algorithms, implemented in Maxent (Phillips & Dudík, 2008). The species distribution modeling was conducted by Maxent (Phillips & Dudík, 2008), with assistance of the R packages dismo (Hijmans *et al.*, 2013). The species occurrence data were extracted from the Global Biodiversity Information Facility (GBIF; http://gbif.org). The continuous probability distributions for each *Lithocarpus* species were generated with 19 BIOCLIM predictors downloaded from WorldClim database (http://www.worldclim.org/bioclim). Trimming and visualization of the continuous probability distributions were conducted by R packages raster (Hijmans & van Etten, 2014). (A)~(H) are the distribution patterns of species with LAEWC, while (I)~(N) are the distributions of species without LAEWC. Dots on the maps are the distribution of specimen records.

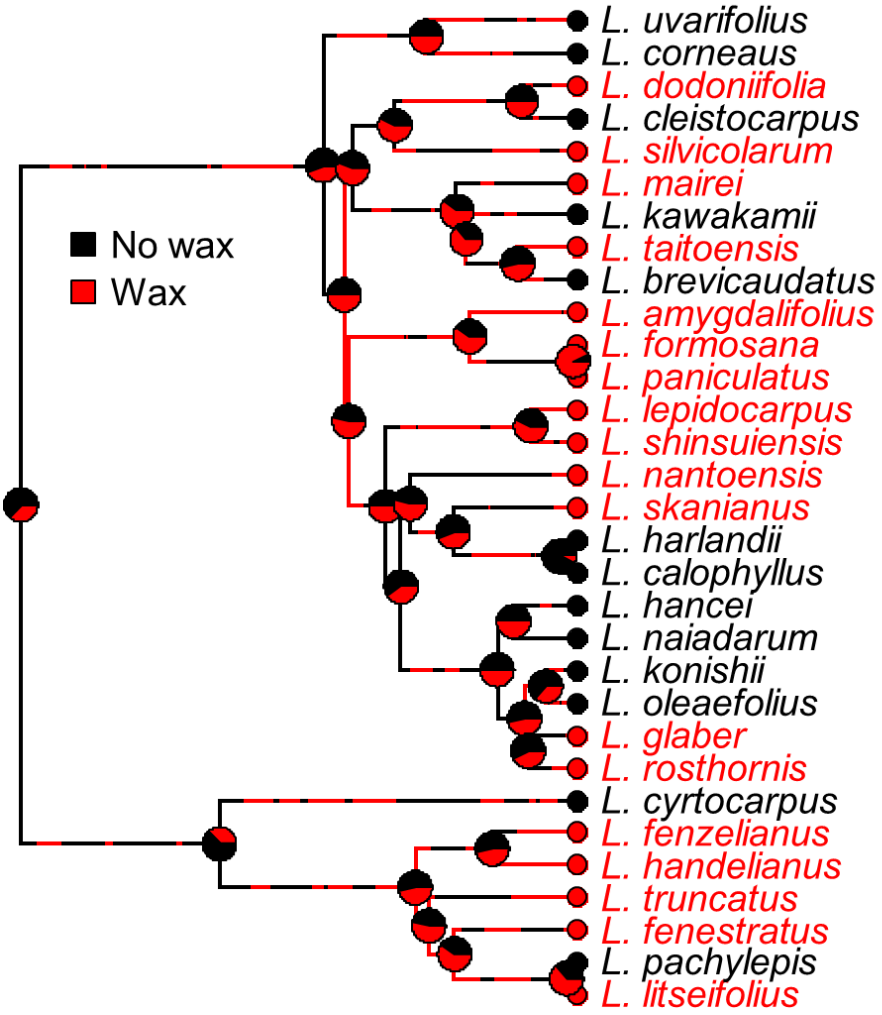

Figure S3. Ancestral state inference of discrete characters using the Maximum likelihood framework assuming one-parameter equal rates (ER) of character transition model and summarize the 300 simulated character reconstructions. This analysis is performed with the function of simmap in R package phytools. Posterior density of ancestral state was presented in pie chart: presence of the leaf abaxial epicuticular wax crystals (LAEWC) is red and absence of LAEWC is black. Colors in the branches show the stochastic character mapping which use an MCMC approach to sample character histories from their posterior probability distribution. We marked the gain and lost events with the red and black arrows, respectively. Fourteen species of this study are marked in blue words. The question marks indicate the ambiguous inference of the ancestral states. This tree is reconstructed based on the chloroplast *atpB*-*rbcL* fragment obtained from the NCBI GenBank using the Yule's pure-birth model. Character state of the LAEWC was determined referred to the Zhou and Xia (Zhou & Xia, 2012). This result indicates ambiguous ancestral states at the basal nodes of the tree with higher probability of absence of LAEWC at subsequent nodes, implying that the earlier ancestors of the extant *Lithocarpus* could be lack of LAEWC. The following "gain" events were observed at certain derived lineages, while certain lineages were suggested to be "lost" the LAEWC again, which inferred that the gain and loss of the LAEWC seemed independently evolving at divergent lineages and the current status of the presence/absence of LAEWC is not symplesiomorphy.

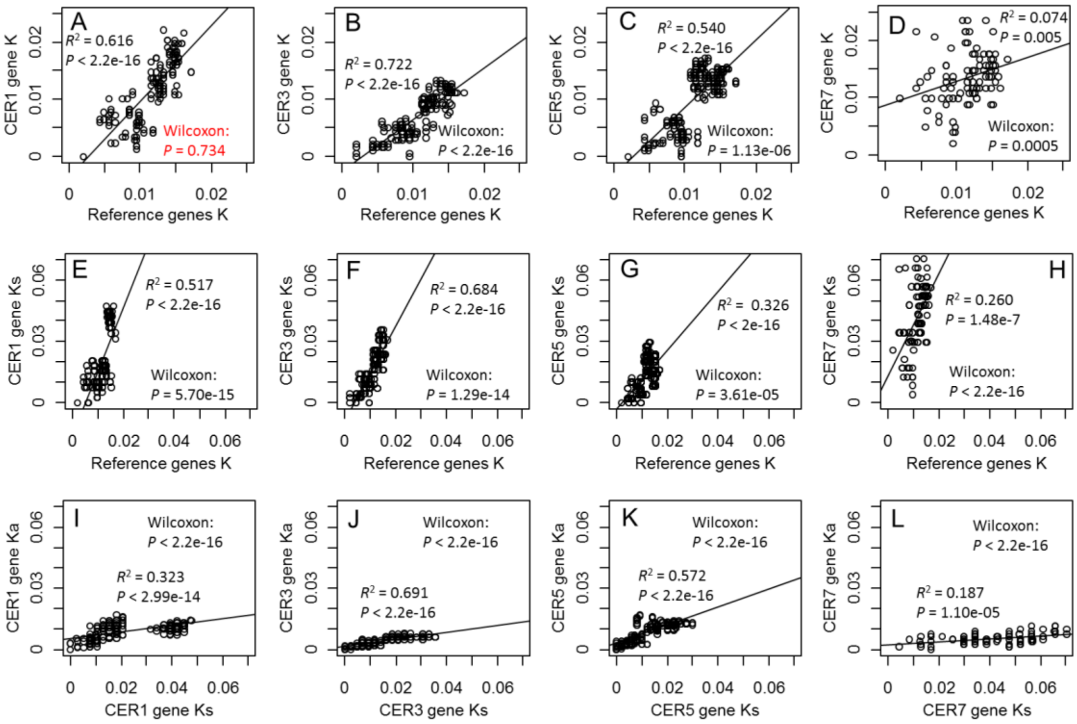

Figure S4. Pairwise comparison of substitution rates (*K*) of LAEWC related genes and reference genes using simple linear regression (SLR) and dependent two-group Wilcoxon Signed Rank Test (WSRT). The R^2 of the SLR is the adjusted R^2. **a~d**, the *K* of LAEWC related gene vs. *K* of reference genes; **e~h**, the synonymous substitution rate (*Ks*) of LAEWC related gene vs. *K* of reference genes; **i~l**, the nonsynonymous substitution rate (*Ka*) vs. *Ks* of LAEWC related gene. The open dots are the observed values of pairwise comparisons and the lines indicate the estimates of simple linear model. Significant results of SLR in all pairs of *K* between the LAEWC related genes and reference genes suggest similar trends of evolution sequence of LAEWC related genes and the species. However, the non-significant WSRT result in *CER1* and reference genes (Supporting information Fig. S4A) indicates the *K* of *CER1* is independent from the rate of species divergence, implies non-neutrally evolving of *CER1*. This inference from this result is consistent with the inference from PAML results (Table 1).

Table S1. Primer list and annealing temperatures used in this study

Locus	Primer	Sequence	Tm(℃)	Accession number	Aligned length (bp)	Percentage of coding regions
SAHH	FagaSAHH-F	CAAGTTCTAACCCTTGAGGATG	52	KY458942 - KY458955	462	100%
	FagaSAHH-R	CTTCTCAGTACCTGTAGTGA				
SAM	FagaSAM-F	CCATTTGCTACTATACTCTTGG	56	KY458956 - KY458961	565	100%
	FagaSAM-R	GGTCTTGATGCTGACAACTGC				
DGD	FagadGd-F	CTCGCTTTCCCTCATCATAGC	58	KY458894 - KY458907	536	48.10%
	FagadGd-R	GGAGGAGCTGGATTCATTGG				
CAP	FagaCAP-F	CTTGCAGTGGGTGTCACTGAG	56	KY458808 - KY458821	587	82.60%
	FagaCAP-R	GCAGGTGTGAGTACATTGGCAG				
ESRK	FagaESRK-F	GATTCTTCGGGGGCTAAAGT	55	KY458908 - KY458926	461	56.80%
	FagaESRK-R	GTGTGAGGGGAGTTGGCGAA				
FAD	FagaFAD-F1	CAGGTTGRAGAACAATGGGTG	52	KY458927 - KY458941	461	100%
	FagaFAD-R1	GCCTGGWGGATTGTTRAGGT				
CER1	CER1-F1	ATGGCTACYAAACCWGGWAT	54	KY458822 - KY458839	1701	100%
	CER1-R1	ATCCCAGCWATACGCCAAGC				
CER3	CER3-F1	ATGGTTGMTWCTTYGTCAGG	53	KY458840 - KY458857	1818	100%
	CER3-R1	ACCRTGTTTTAATGCWGCTTC				
CER5	CER5-F1	ACASTATGGAGATWGAGRTAGCT	52	KY458858 - KY458879	2058	100%
	CER5-R1	TGCTTCTAGTGGAGAGGAG				
CER7	CER7-F1	CGTTTAACAGTGAACGAGAAG	55	KY458880 - KY458893	1032	100%
	CER7-R3	CCTCCACTRGTGARAGATG				

Aligned length: the amplified and aligned length of each locus

Table S2. Results of likelihood ratio test of hypotheses of positive selection on retaining the gain or loss of LAEWC trait. The result shows that all LAEWC related genes were not positively selected for retaining the gain of LAEWC or retaining the loss of LAEWC. These three hypotheses were tested under the branch model implemented by PAML with labeling "**$**" on foreground branches, which indicates allowing ω > 1 on all deriving branches of the marked foregrounds. The results showed that none of null hypotheses can be rejected by the alternative hypotheses in all four LAEWC related genes.

Hypothesis [a]	Gene	$\ln L_0$ [b]	$\ln L_A$ [b]	$2\Delta L$	df	P
Hypothesis 1'	*CER1*	-2969.330	-2970.308	-1.963	1	NA
	CER3	-2923.210	-2922.587	1.245	1	0.265
	CER5	-3516.910	-3516.750	0.326	1	0.568
	CER7	-2009.030	-2009.013	0.028	1	0.866
Hypothesis 2'	*CER1*	-2969.330	-2969.950	-1.246	1	NA
	CER3	-2923.210	-2923.153	0.113	1	0.737
	CER5	-3516.910	-3516.910	0.005	1	0.943
	CER7	-2009.030	-2008.363	1.329	1	0.249
Hypothesis 3'	*CER1*	-2969.330	-2968.744	1.166	2	0.558
	CER3	-2923.210	-2923.126	0.167	2	0.920
	CER5	-3516.910	-3516.533	0.760	2	0.684
	CER7	-2009.030	-2008.317	1.422	2	0.491

[a] Hypotheses modified from Fig. 1 and Table 1, but the foreground branches are changed to all deriving branches of the marked branches.

[b] $\ln L_0$: natural logarithm of the likelihood of null model; $\ln L_A$: natural logarithm of the likelihood of alternative mode

Table S3. Ecophysiological measurements and altitudinal distribution of *Lithocarpus* species tested in this study

Species	LAEWC	N(YII)	YII±STD	PA	$\delta^{13}C$	$\delta^{15}N$	C	N	C/N	Min Alt	Max Alt	ΔAlt
L. amygdalifolius	waxy	40	0.7709±0.0267	47.70	-31.076	-0.856	0.550	0.015	36.552	250m	2000m	1750m
L. brevicaudatus	non-waxy	20	0.6814±0.0181	44.11	-31.018	-0.862	0.499	0.017	29.961	200m	2350m	2150m
L. cornea	non-waxy	20	0.6995±0.0382	72.68	-30.654	-0.510	0.484	0.020	24.170	100m	1400m	1300m
L. dodonaeifolius	waxy	20	0.7457±0.0326	44.53	-31.254	-1.618	0.550	0.014	38.397	350m	1600m	1250m
L. formosanus	waxy	20	0.7006±0.0206	45.33	-31.833	-1.393	0.504	0.014	36.320	100m	550m	450m
L. glaber	waxy	20	0.7376±0.0273	28.65	-30.451	-0.499	0.499	0.020	24.504	450m	1050m	600m
L. hanceii	non-waxy	20	0.7438±0.0245	26.72	-33.455	-0.964	0.503	0.031	16.110	100m	2700m	2600m
L. harlandii	non-waxy	20	0.7266±0.0255	30.00	-31.962	-1.589	0.482	0.015	32.745	350m	600m	250m
L. kawakamii	non-waxy	20	0.7345±0.0403	34.03	-32.413	-0.512	0.491	0.026	18.893	350m	2350m	2000m
L. konishii	non-waxy	52	0.6646±0.0541	52.87	-30.841	1.125	0.483	0.020	24.220	100m	1150m	1050m
L. lepidocarpus	waxy	40	0.7702±0.0245	61.07	-31.334	-0.606	0.529	0.026	20.297	600m	2230m	1630m
L. nantoensis	waxy	20	0.6290±0.0250	83.09	-31.844	-2.622	0.481	0.017	29.146	550m	1300m	750m
L. shinsuiensis	waxy	20	0.7451±0.0315	103.10	-30.893	-1.509	0.495	0.013	38.697	300m	1200m	900m
L. taitoensis	waxy	20	0.7236±0.0468	36.46	-32.009	-0.763	0.521	0.018	28.848	700m	1300m	600m

LAEWC: leaf abaxial epicuticular wax crystals
N(YII): sample size of YII measurements
YII±STD: phytochemical yield of photosystem II and the standard deviation
PA: contents of phenolic acids
Min Alt, Max Alt, andΔAlt: the lowest, the highest, and the range of altitudinal distribution
